# Predictors of short-term response and the role of heavy alcohol use in treatment of depression

**DOI:** 10.1186/s12888-023-05366-8

**Published:** 2023-11-27

**Authors:** Kaisa E. Luoto, Antero Lassila, Esa Leinonen, Olli Kampman

**Affiliations:** 1 Department of Psychiatry, Faculty of Medicine and Health Technology, Tampere University, Tampere, Finland; 2 Department of Psychiatry, Tampere University Hospital, The Wellbeing Services County of Pirkanmaa, Tampere, Finland; 3 Department of Psychiatry, Seinäjoki Central Hospital, The Wellbeing Services County of South Ostrobothnia, Seinäjoki, Finland; 4 Department of Clinical Sciences, Psychiatry, Umeå University, Umeå, Sweden; 5 Department of Clinical Medicine (Psychiatry), Faculty of Medicine, University of Turku, Turku, Finland; 6 Department of Psychiatry, The Wellbeing Services County of Ostrobothnia, Vaasa, Finland

**Keywords:** Depression, Alcohol Use Disorder, Outcome

## Abstract

**Background:**

Depression and alcohol use disorders frequently co-occur. However, research on psychosocial interventions for treating this dual pathology is limited. The Ostrobothnian Depression Study (ODS) aimed to increase the systematic use of evidence-based methods, particularly among patients with comorbid depression and substance use in a naturalistic setting. This is a secondary analysis of the ODS study. The aim of the present study was to explore the predictors of a response to treatment during the first six months of the ODS intervention with a specific focus on the role of comorbid heavy alcohol use.

**Methods:**

The study sample (*n* = 242) comprised psychiatric specialist care patients with depression (Beck Depression Inventory score ≥ 17) at baseline. Patients with a baseline Alcohol Use Disorders Identification Test (AUDIT) score > 10 (*n* = 99) were assigned to the AUD (Alcohol Use Disorder) group in this study. The ODS intervention comprised behavioral activation (BA) for all and additional motivational interviewing (MI) for those in AUD group. The predictors of response to treatment (minimum of 50% reduction in depressive symptoms) during the first six months were analyzed with logistic regression models.

**Results:**

In the total sample at six months (*n* = 150), predictors of response to treatment were more severe depression (OR 1.10, CI 1.02–1.18), larger amounts of alcohol consumed (OR = 1.16, CI 1.03–1.31) and antipsychotic medication “not in use” (OR = 0.17, CI 0.07–0.44). In the non-AUD group (*n* = 100), more severe depression (OR 1.12, CI 1.01–1.25) and antipsychotics “not in use” (OR 0.20, CI 0.06–0.67) also predicted a positive response. Among AUD group patients (*n* = 50), larger amounts of alcohol consumed (OR 1.54, CI 1.04–2.27) and antipsychotic medication “not in use” (OR 0.12, CI 0.02–0.60) predicted a response to the treatment intervention.

**Conclusions:**

The severity of symptoms and comorbid disorders were found to predict better treatment response, suggesting that the intervention was more effective in patients with severe symptoms. Patients with depression should be treated effectively regardless of having concomitant AUD. The results of this study suggest that BA combined with MI should be one of the treatment options for this dual pathology.

**Trial registration:**

ClinicalTrials.gov Identifier NCT02520271 (11/08/2015).

## Background

Depressive disorders cause major suffering and disability globally [1]. Psychiatric treatment options are fortunately evolving and the stigma surrounding the treatment of depression, as well as other mental disorders, is diminishing [2, 3]. However, there is a clear need for more personalized treatment options as well as for more active use of a variety of therapeutic approaches [4]. At the same time, the limited treatment resources in psychiatric care should be used more efficiently.

Co-occurring disorders with depression are common in psychiatric patients both in primary care and in specialist care [5–7]. Approximately half of patients with depression have a comorbid anxiety disorder, and various personality disorders as well as alcohol and other substance use disorders are common [8, 9]. Comorbidities lead to treatment resistance and more severe symptoms compared to single diagnosis conditions [8, 10, 11]. Co-occurring disorders also have an effect on the choice of the most suitable form of treatment [12, 13].

Many factors influence the outcome of depression treatment. For example, the type of treatment (therapy, pharmacotherapy, neuromodulation), the amount and duration of treatment, the severity of symptoms at baseline, other psychiatric disorders, the previous course of the illness and individual genetic vulnerability factors [14]. For example, the presence of chronic medical conditions or pain may also negatively influence patients’ treatment outcome [15, 16].

There are many effective treatments for depression, including various medications and psychosocial interventions [17, 18]. However, studies of treatment options specifically in real-world settings are scarce. For example, patients with comorbid alcohol use disorders (AUD) are often excluded from depression trials even though the problematic use of alcohol is one of the most common comorbidities in depressive patients [19–21]. Patients with this dual pathology suffer from a more severe symptom profile than those without AUD, and the probability of recovery has likewise been found to be associated with the extent of alcohol use [22–24]. It would be important for treatment to be able to address both disorders simultaneously.

There is a growing body of evidence regarding the use of various pharmacotherapeutic options in treating simultaneous depression and AUD [25–27]. A recent study of Hunt et al. [28] explored the conventional wisdom that heavy alcohol consumers do not necessarily engage well with or benefit from psychological treatments for depression and anxiety. The researchers found no confirmation of this, but concluded that those with a high alcohol consumption benefit from treatment the same as others. There are also psychotherapeutic interventions available for the treatment of comorbid depression and AUD [26, 29]–[31]. However, so far the evidence does not support any intervention over others for this comorbidity [32–34].

The Ostrobothnian Depression Study (ODS; ClinicalTrials.gov Identifier: NCT02520271, 11/08/2015) aimed to increase the systematic use of evidence-based methods in psychiatric care and particularly among patients with comorbid depression and heavy alcohol use. The idea was to use relatively simple and firmly evidence-based methods which can be implemented in the existing treatment facilities in psychiatric specialist care. The ODS treatment intervention comprised Behavioural Activation (BA) [35] for all and additional motivational interviewing (MI) [36] for patients with heavy alcohol use at the beginning of the treatment.

In the context of psychiatric care, it is essential that the interventions used are feasible for a heterogeneous patient population and easy to implement. This is the rationale behind the choice of these methods for the ODS intervention. A separate implementation study has been reported earlier [37]. BA is considered a well-established treatment for depression and is also emerging as an option for the treatment of substance use disorders [35, 38]. Research on BA in the treatment of comorbid depression and alcohol use problems is so far rather limited, but the existing results are promising [39, 40]. MI is an effective method for treating substance use disorders [36, 41].

We have previously reported results concerning improvements in functional ability and health-related quality of life in patients treated with the ODS intervention [42, 43]. We found that the patients’ functional ability improved more during the treatment than the control patients who received treatment as usual. We also found that patients’ quality of life improved significantly at the first stages of treatment, regardless of whether or not they had heavy alcohol consumption.

The present study reports a secondary analysis of the results of ODS intervention. We explored the factors that would predict depression treatment response when behavioral activation is used for a heterogeneous population of psychiatric care patients. It would be important to be able to establish the most appropriate treatment for each patient as early as possible [13]. The study patients have a variety of comorbid disorders at baseline, and some also have heavy alcohol use. The basic assumption was that even such a heterogeneous population could be treated with relatively simple but evidence-based brief interventions.

The specific aim of the present study was to explore the predictors of response to the ODS treatment approach in short-term follow-up of six months. We focused especially on the impact of alcohol use on the outcome of treatment for depression.

## Materials and methods

### Patient sample

Patients (*n* = 242) were recruited from the specialist care outpatient clinics and one inpatient ward in what was then the South Ostrobothnia Hospital District, Finland during the period 2009–2013. The main inclusion criterion was Beck Depression Inventory (BDI, version 1 A) score ≥ 17. Only patients with a suspected or verified psychotic disorder or organic brain disease were excluded. Age range was 18–64 (61.2% female). The basic characteristics of the patient sample and the baseline measures are presented in Table 1.

**Table 1:** Basic characteristics and baseline measurements of the Ostrobothnian Depression Study patients

	*n (%)*
Number of patients	242
Female / Male	148 (61.2) / 94 (38.8)
Outpatient / Inpatient	189 (78.1) / 53 (21.9)
*Register based* ^*a*^ *clinical diagnoses (ICD-10) as determined in the patient´s records*	
Alcohol dependence (current)	47 (19.4)
Use of other substance (last 12 months)	22 (9.1)
*Primary psychiatric diagnosis in the patient’s record*	
Depressive disorder (F32.x)	98 (40.5)
Recurrent depressive disorder (F33.x)	92 (38.0)
Bipolar disorder (F31.x)	19 (7.9)
Anxiety disorders (F40-43.x)	9 (3.7)
Other disorders	18 (7.4)
*Secondary psychiatric diagnosis in the patient’s record*	
Panic disorder (F41.x)	26 (10.7)
Phobic and other anxiety disorders (F40,42,43.x)	22 (9.1)
Personality disorder (F6x.x)	9 (3.7)
	***mean (SD)***
Age (years)	38.8 (12.2)
Baseline MADRS^b^	23.2 (6.7)
Baseline BDI^c^	27.9 (7.3)
Baseline AUDIT-C^d^	7.6 (2.1)
Baseline GAF^e^	45.9 (10.7)

^a^Register based diagnoses determined by the doctor in charge in previous treatment contacts

^b^Montgomery–Åsberg Depression Rating Scale

^c^Beck Depression Inventory

^d^Alcohol Use Disorders Identification Test, consumption subscale

^e^Global Assessment of Functioning scale

Patients with baseline Alcohol Use Disorder Identification Test (AUDIT) > 10 were categorized as having an alcohol use disorder (AUD group) in this study (n = 99, 40.9%, 61 males). For clarity, we decided to refer to the group of heavy drinkers as the AUD group, although not all of them would have met the diagnostic criteria for ICD-10 alcohol use disorder. The AUDIT cut-off value chosen exceeds the risk consumption limit (> 8) commonly used in the literature [44, 45].

Antidepressive medication was prescribed for 206 (85.1%) patients (cumulative average dose 28.0 mg, SD 20.6 mg fluoxetine equivalents) and antipsychotic medication for 66 (27.3%) patients (cumulative median dose 62.5 mg, IQR 93.75 chlorpromazine equivalents). Patients were on different medications at the time of admission. Medication was changed during the ODS intervention as needed. The main responsibility for medication management remained with the clinician, who was in charge of the patient’s overall treatment. The medication was reviewed and, if necessary, adjusted by the study doctor at the beginning of the ODS intervention.

### ODS intervention

Of the patients included in the study, those with high alcohol consumption at baseline (AUD group) were first given MI. However, all patients were treated with BA for depression, regardless of their alcohol use. Unfortunately, only a small proportion of therapists reported more detailed information on how well the chosen methods were implemented [46].

**Fig. 1:**
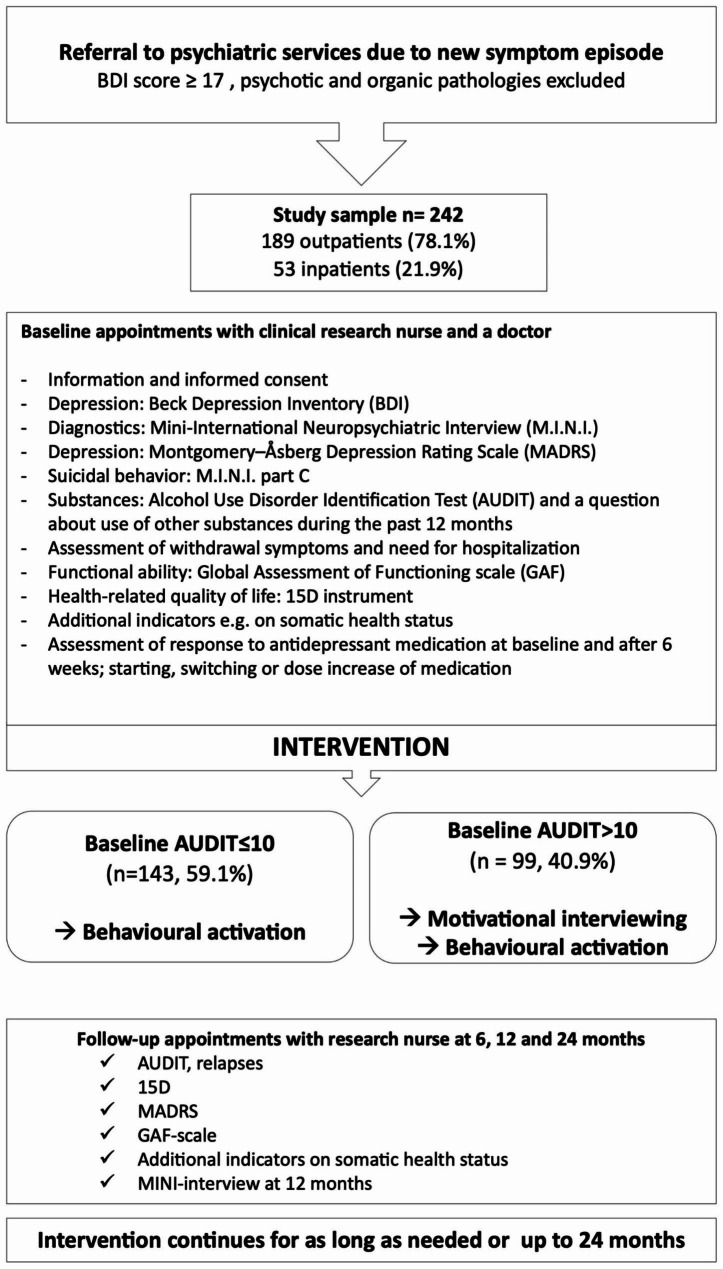
The Ostrobothnian Depression Study protocol

Figure 1 shows the ODS protocol in detail and the methodology have also been reported elsewhere [42, 43, 47].

### Measures

Various clinical outcome measures were obtained at six-month, 12-month, and 24-month follow-up points. For the purposes of this paper, the key measures were The Montgomery-Åsberg Depression Rating scale (MADRS) [48], AUDIT [49] and the Alcohol Use Disorders Identification Test Concise (AUDIT-C) [45].

MADRS was used to assess the symptoms of depression during the follow-up. All ratings were carried out by study nurse or study doctor and they were not blinded concerning study objectives. The response to treatment was defined as a minimum 50% decline in Montgomery–Åsberg Depression Rating Scale (MADRS) score compared to baseline. A 50% reduction in symptom severity is generally considered the standard of response to treatment for depression [50].

AUDIT was used to assess the amount of alcohol consumed at baseline and at follow-up, the AUDIT-C was also collected. AUDIT-C consists of the first three questions of AUDIT and specifically assesses the amount and frequency of alcohol consumption. Both indicators are validated screening tools for heavy alcohol consumption [45, 49, 51]. The cut-off value chosen for the AUD group exceeds the risk consumption limit commonly used in the literature (> 8) [44, 45]. Neither measure is a direct diagnostic tool for AUD.

#### Drop-out

**Fig. 2:**
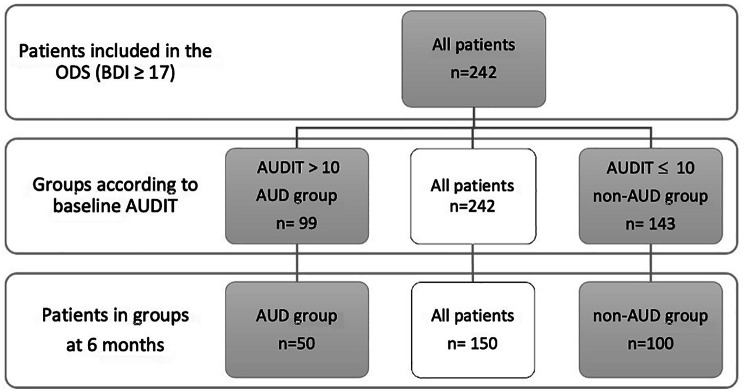
A diagram illustrating participant drop-out during the first six months of Ostrobothnian Depression Study (ODS)

Figure 2 shows the drop-out rate of patients during the first six months of follow-up. Nine patients (6.3%) in the non-AUD group and 11 patients (11.1%) in the AUD group discontinued treatment during the six-month follow-up due to recovery, while 20 patients (14.0%) in the non-AUD group and 31 patients (31.3%) in the AUD group discontinued treatment for unknown reasons. There were four deaths in the intervention group (mortality rate 1.7%). We have no data on specific causes of death.

#### Statistical methods

This is a secondary analysis of the ODS study. The sample analysed for the present study was of those participants (baseline *n* = 242) of the experimental arm of the parent study. Analyses were conducted for the sample of patients for whom data were available for all variables used at six months (*n* = 150), and separately for the remaining non-AUD (*n* = 100) and AUD patients (*n* = 50) in this sample. This was due the different interventions between the sub-groups (AUD patients initially received additional MI).

The Firth´s logistic regression was used to analyze the factors predicting response to treatment of depression during the first six months of follow-up. Covariates in all models run were age, gender, baseline MADRS, baseline AUDIT-C, cumulative daily dose of antidepressive medication, regular use of antipsychotic medication, and baseline diagnosis of any anxiety disorder.

Before the regression analysis, we checked that the correlations between the variables were r < 0.5. The Alcohol Use Disorders Identification Test - Concise (AUDIT-C) containing the first three questions of the AUDIT questionnaire on consumption and frequency was used in this analysis instead of AUDIT since it elicits more specifically the amount of alcohol consumed.

Calculations were performed with SPSS for Apple Macintosh versions 24 and 25 and Stata Statistics and Data Science (Copyright 1985–2021 StataCorp LLC, https://www.stata.com).

## Results

AUDIT scores of patients in the AUDIT group (*n* = 99) decreased during the first six months of follow-up from 20.8 (SD 7.2) at baseline to 11.8 (SD 6.0) at six months of follow-up (p < 0.001). AUDIT-C score was available for 49 (49.5%) AUD-group patients at six months. In this patient group, AUDIT-C decreased from 7.55 (SD 2.07) at baseline to 6.16 (SD 2.83) at six months (p = 0.005 and Cohen´s d = 0.79).

Table 2 describes the age and baseline symptom scores of the patients (*n* = 150) included in the 6-month follow-up compared to the whole patient population (*n* = 242).

**Table 2:** Comparison of baseline and 6-month follow-up patients in terms of baseline measurements

	*n = 242 (baseline)*	*n = 150 (included in 6 months follow-up)*	*n = 92 (lost at 6 months follow-up)*	*p* ^*a*^
	*mean (SD)*	*mean (SD)*	*mean (SD)*	
Age (years)	38.8 (12.2)	39.4 (12.0)	37.7 (12.5)	0.301
Baseline MADRS^b^	23.2 (6.7)	23.2 (5.8)	23.2 (8.2)	0.966
Baseline BDI^c^	27.9 (7.3)	27.5 (7.0)	28.4 (7.7)	0.355
Baseline AUDIT-C^d^	7.6 (2.1)	4.3 (3.2)	5.5 (3.6)	0.004
Baseline GAF^e^	45.9 (10.7)	46.3 (9.9)	45.1 (9.9)	0.422

^a^p-value for difference between groups included or lost at 6 months (independent samples t-test)

^b^Montgomery–Åsberg Depression Rating Scale

^c^Beck Depression Inventory

^d^Alcohol Use Disorders Identification Test, consumption subscale

^e^Global Assessment of Functioning scale

During the first 6 months, those who dropped out of treatment had higher alcohol consumption at baseline than those who stayed in treatment (p = 0.004). In analysing the change in MADRS between baseline and six months follow-up of the 150 patients (who provided data at month 6) the mean change in MADRS was 10.0 points (SD 9.9). In total, 67 (44%) patients showed a 50% reduction in MADRS total score. The corresponding numbers in those without AUD (*n* = 100) and in those with AUD (*n* = 50) were 44 (44.0%) and 23 (46.0%), respectively (p = 0.82). The detailed results of the Firth´s logistic regression models analyzing the factors predicting a response to treatment during the first six months are presented in Table 3.

**Table 3:** Logistic regression for the factors predicting response^1^ to treatment of depression during the first 6 months of follow-up in the Ostrobothnian Depression Study. Logistic regression was conducted for the total sample and separately for patients with or without heavy alcohol use (AUD)^2^.

	All patients (*n* = 150)		Patients without AUD (*n* = 100)		Patients with AUD (*n* = 50)	
	Nagelkerke R Square 0.250		Nagelkerke R Square = 0.257		Nagelkerke R Square = 0.582	
	**OR (95% CI)**	**p**	**OR (95% CI)**	**p**	**OR (95%CI)**	**p**
Age	1.02 (0.98–1.05)	0.333	1.00 (0.96–1.04)	0.933	1.06 (0.99–1.14)	0.093
Gender^3^	0.51 (0.23–1.12)	0.094	0.56 (0.18–1.74)	0.320	0.41 (0.09–1.94)	0.263
Baseline MADRS	1.10 (1.02–1.18)	0.008	1.12 (1.01–1.25)	0.027	1.11 (0.98–1.25)	0.093
Baseline AUDIT-C^4^	1.16 (1.03–1.31)	0.013	1.11 (0.89–1.38)	0.371	1.54 (1.04–2.27)	0.029
Dose of antidepressants^5^	0.99 (0.97–1.01)	0.172	0.98 (0.95-1.00)	0.066	1.01 (0.98–1.05)	0.493
Use of antipsychotics^6^	0.17 (0.07–0.44)	< 0.001	0.20 (0.06–0.67)	0.010	0.12 (0.02–0.60)	0.011
Any anxiety disorder	0.50 (0.22–1.14)	0.099	0.40 (0.15–1.08)	0.070	1.32 (0.30–5.87)	0.712

^1^ Positive response was defined as minimum of 50% reduction in Montgomery-Åsberg Depression Rating Scale (MADRS) score

^2^Groups formed according to alcohol use at baseline according to the Alcohol Use Disorders Identification Test, AUDIT: non-AUD group when baseline AUDIT ≤ 10 and AUD group when baseline AUDIT > 10

^3^Men compared to women

^4^Alcohol Use Disorders Identification Test, first three questions eliciting quantity-frequency

^5^Cumulative dose in fluoxetine equivalents. The OR < 1 indicates a greater likelihood for response in patients with a lower dose of antidepressant

^6^The OR < 1 indicates a greater likelihood for response in patients not on antipsychotic medication

In the total sample at six months (*n* = 150), predictors of response to treatment were higher baseline MADRS score indicating more severe depression (OR 1.10, CI 1.02–1.18), higher baseline AUDIT-C score indicating larger amounts of consumed alcohol (OR 1.16, CI 1.03–1.31), and antipsychotic medication “not in use” (OR 0.17, CI 0.07–0.44; OR < 1 indicates a greater likelihood for response in patients not on antipsychotic medication).

In the non-AUD group (*n* = 100) higher MADRS score at baseline (OR 1.12, OR 1.01–1.25) and antipsychotic medication “not in use” (OR 0.20, OR 0.06–0.67) predicted response. In patients with comorbid AUD (*n* = 50), higher baseline AUDIT-C score (OR 1.54, OR 1.04–2.27), and antipsychotic medication “not in use” (OR 0.12, CI 0.02–0.60) predicted a response to the treatment intervention.

## Discussion

Many factors affect the prognosis of depression, e.g., symptom severity, co-occurrence of other psychiatric disorders and substance use disorders. The present study investigated predictive factors for short-term treatment response in patients treated for depression in psychiatric care. The focus was especially on the impact of comorbid heavy alcohol use on treatment outcome. The findings of the present study are encouraging; patients with more severe symptoms of depression and heavier alcohol use (and thus with somewhat poorer prognosis) recovered well with this intervention.

The data on the overall response rate to psychotherapies shows that psychotherapy is better than treatment as usual or wait list conditions [52]. Furthermore, the combined use of psychotherapy and pharmacotherapy was found to be more effective than either one alone in the treatment of depression [17]. For these reasons, evidence-based psychotherapeutic methods should be used systematically in the treatment of depressive disorders.

In our sample, more severe depressive symptoms predicted a response to treatment among total sample and among non-AUD patients. In every patient group, antipsychotic medication “not in use” predicted a better response to treatment. These results may indicate that positive treatment response was related to severe depression, which however, was managed without antipsychotics. Higher dose of antidepressant and a need for antipsychotic augmentation in depression are often related to more severe or complicated symptoms [53]. For example, antipsychotics may be used for the treatment of comorbid anxiety or severe problems with sleep. However, the dose of medications is not a direct measure of the severity of depression. Dosage can be related to many variables such as a person’s size, metabolism, history of antidepressant use and the type of antidepressant prescribed, not just the severity of symptoms.

Even though both depressive and alcohol use disorders are common, data on the efficacy of treatment interventions for this dual pathology is scarce [33, 34]. It is therefore important to study the effectiveness of various interventions in this patient population. In the ODS data, it was possible to examine the impact of heavy alcohol use on outcomes. Groups were defined using AUDIT scores > 10 instead of diagnostic criteria for alcohol misuse. The rationale was that the current AUDIT score better reflects the level of recent alcohol use, the impact of which on treatment was to be investigated.

The baseline depressive symptoms may be influenced by depressive symptoms due to heavy drinking. Reducing alcohol consumption in itself alleviates symptoms of depression [54]. Treatment of depression co-occurring with alcohol use disorder has been found to be associated with significant early improvement in the depressive symptoms, regardless of whether the depression is considered to be independent or due to heavy drinking [55]. In this sample, the level of alcohol consumption at the beginning of treatment predicted a positive response in the total sample and in the AUD group. In non-AUD patients the AUDIT-C score was not relevant since consumption was already low.

Among the AUD group, AUDIT-C decreased significantly at the beginning of the intervention. This is probably related to the structured use of MI in the first part of the ODS intervention. It is not possible to distinguish between the effect of MI and BA in this group of patients. Both contain elements of the same nature, such as helping patients to reflect on their own behaviour and motivating them to make changes [56]. The use of structured MI in the early stages may be a key part of the outcome of the intervention for patients in the AUD group.

Despite the various possible treatment interventions for depression, comorbid AUD tends to cause at least partly unnecessary treatment pessimism [17, 28, 57]. As discussed in earlier research, excessive alcohol use is just one predictor for a poorer outcome in the treatment of depression, as comorbid disorders tend to cumulate [23, 58]. The assumption that substance use may cause inability to benefit from psychiatric treatments may be related to the stigma which these dual diagnosed patients commonly face [59]. On the other hand, heavy alcohol consumption can complicate treatment adherence, which was also seen in this study.

The strength of this study was its population, which represents well the depressive patients treated in psychiatric specialist care. It is important to study treatment interventions in a naturalistic setting, where patients with depression usually have a variety of comorbidities affecting treatment outcomes. In RCTs, the patient population tends to be less heterogeneous, and those with substance use disorders in particular are excluded from the study data. This study contributes to our knowledge of variables related to depression treatment outcome among non-selected psychiatric care patients. As this was a short-term intervention, and the initial phase of treatment was the most intensive, we decided to focus on predicting short-term outcomes.

The limitations of this study include the relatively small patient population, especially in the subgroup analyses, which may limit the generalizability of the results. The skewness in the gender distribution between the AUD and non-AUD groups and the drop-out must also be considered. Those who dropped out in the first six months had higher AUDIT-C scores on admission than those who stayed on treatment. While the heterogeneity of the patient population is a particular strength of the study, it also poses challenges for the interpretation of the results.

In this study design, it is not possible to distinguish the role of medication in alleviating depressive symptoms. Patients’ antidepressant medications varied and medication was adjusted as needed. This is an important limitation that impacts the generalizability of results as they pertain to predictors of BA outcome and should be noted as such. However, in this respect, the setting also corresponds to the setting of the actual clinical work.

The choice of any anxiety disorder as a covariate was based on the fact that these are very common in patients with depression and typically affect treatment outcomes [60, 61]. However, not all common co-occurring disorders could be taken into account in this study. For example, no data on personality disorders diagnosed by structured interview were available in this dataset [62, 63]. In the diagnoses recorded in the patient’s file, the prevalence of personality disorders was lower than expected [64]. Therefore, it was not possible to include them in the analyses, which is a clear limitation.

As all patients included in the study were depressive at baseline, depression associated with bipolar disorder was not distinguished from unipolar depression in this study. A diagnosis of bipolar disorder was not considered an essential confounder for the treatment of current symptoms of depression with the selected methods.

The data on socio-economic status were not taken into account in the conducted analyses. The study did not systematically collect data on patients’ income levels. Due to the limited sample size we had to restrict the number of variables used in multivariate analyses. We focused primarily on the clinical variables and we had to leave out the socioeconomical variables.

We tested the likelihood of the results being due to the effects of the regression to mean phenomenon with separate analyses (data not shown). However, there seemed be no biased results when analysed according to responders, dropped out patients or depression severity leading to a conclusion that the present results are not likely affected by regression to mean.

Those with high or low alcohol consumption at the beginning of treatment have been separated into their own groups, but no comparison has been made between these groups. Patients in the AUD group received MI in the early phase of treatment, but there is a lack of data on its implementation. For this group, it is therefore not possible to distinguish the effect of BA and MI on treatment outcome.

## Conclusions

Behavioural activation was used to treat patients with depression and other co-existing psychiatric disorders or heavy alcohol use at baseline. Those with heavy alcohol consumption at the beginning were first given motivational interviewing. The data show that severity of disease and comorbidity are substantial predictors of better outcomes, suggesting that BA and MI are more effective in severely affected patients. This relatively straightforward evidence-based psychosocial intervention implemented in everyday clinical work may be beneficial in preventing treatment resistant depression or chronicity. It should be noted that depressive patients with comorbid heavy alcohol use benefit from psychosocial interventions and should not be left without treatment. Addressing alcohol consumption immediately at the beginning of treatment may increase patients’ chances of benefiting from depression treatment.

## Data Availability

The datasets generated and/or analyzed during the current study are available from the corresponding author on reasonable request.

## List of Abbreviations

AUD: Alcohol Use Disorder
ODS: Ostrobothnian Depression Study
BDI: Beck Depression Inventory
MADRS: Montgomery–Åsberg Depression Rating Scale
AUDIT: Alcohol Use Disorder Identification Test
AUDIT-C: Alcohol Use Disorders Identification Test Concise
BA: Behavioral Activation
MI: Motivational Interview
GAF: Global Assessment of Functioning scale
